# Lose the Stress: Viscoelastic Materials for Cell Engineering

**DOI:** 10.1016/j.actbio.2022.03.058

**Published:** 2022-04-08

**Authors:** Emily M. Carvalho, Sanjay Kumar

**Affiliations:** a Department of Chemical and Biomolecular Engineering, University of California, Berkeley, Berkeley, CA 94720, USA; b University of California, Berkeley–University of California, San Francisco Graduate, Program in Bioengineering, Berkeley, CA 94720, USA; c Department of Bioengineering, University of California, Berkeley, Berkeley, CA 94720, USA; d Department of Bioengineering and Therapeutic Sciences, University of California, San Francisco, San Francisco, CA 94158, USA

**Keywords:** Viscoelastic, collagen, alginate, hyaluronic acid, electrospun fibers, cell migration, mechanobiology, stress relaxation

## Abstract

Biomaterials are widely used to study and control a variety of cell behaviors, including stem cell differentiation, organogenesis, and tumor invasion. While considerable attention has historically been paid to biomaterial elastic properties, it has recently become clear that viscous (loss) properties can also powerfully influence cell behavior. Here we review advances in viscoelastic materials for cell engineering. We begin by discussing collagen, an abundant naturally occurring biomaterial that derives its viscoelastic properties from its fibrillar architecture, which enables dissipation of applied stresses. We then turn to two other naturally occurring biomaterials that are more frequently modified for engineering applications, alginate, and hyaluronic acid, whose viscoelastic properties may be tuned by modulating network composition and crosslinking. We also discuss the potential of exploiting engineered fibrous materials, particularly electrospun fiber-based materials, to control viscoelastic properties. Finally, we review mechanisms through which cells process viscous and viscoelastic cues as they move along and within these materials. The ability of viscoelastic materials to relax cell-imposed stresses can dramatically alter migration on two-dimensional surfaces and confinement-imposed barriers to engraftment and infiltration in three-dimensional scaffolds.

## Graphical Abstract

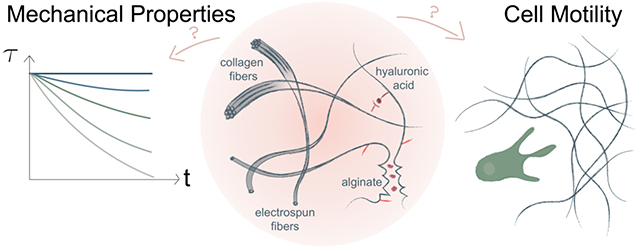

## 1. Introduction

Polymer hydrogel biomaterials are widely employed in tissue engineering, disease modeling, and mechanistic studies. Over the past two decades, it has become clear that scaffold mechanical properties can strongly influence many key cell behaviors, including migration, differentiation, and morphogenesis [1–5]. This important body of work has drawn closer attention to the mechanics of the biomaterial used for a given application. For example, it is increasingly appreciated that traditional culture surfaces such as glass and tissue-culture polystyrene are substantially stiffer than nearly all tissues in the body, which may produce unintended consequences when modeling or recapitulating physiological processes.

The vast majority of effort to engineer hydrogel mechanics has focused on elastic properties, i.e. the ability of the scaffold to store applied stresses. However, most tissues are viscoelastic and capable of dissipating as well as storing stress, and recent studies have revealed the importance of this dual character in controlling cell and tissue behavior, such as in the tendons, skin, and brain [6, 7]. These studies have revealed the importance of viscoelasticity not only in normal physiological processes, but also in disease progression. As a result, increased effort has been devoted to incorporating viscous (loss) properties into biomaterials for engineering applications. In native biomaterials, stress relaxation is closely tied to material microstructure. For example, the fibrillar architecture of collagen-based tissue enables dissipation of stress through rearrangement of the fibers [6]. As another example, brain matrix derives its viscoelastic properties from the high water content and noncovalent crosslinking of its hyaluronic-acid-rich network [8].

Hydrogels can also be tuned to mimic the viscoelastic nature of native tissue or introduce specific properties to study a given process or phenomenon. Some biomaterials are commonly crosslinked noncovalently, such as collagen, alginate, and electrospun fibers, and thus may dissipate stress through force-induced remodeling of the network. This stress-relaxing behavior is uncommon in covalently crosslinked networks, where the crosslinks are not easily broken by force. Therefore, stress-relaxation properties can be further tuned through a variety of chemical modifications, including crosslinks that may be broken under force and then reformed [9–16]. Accordingly, these modulations also enable fabrication of slow stress-relaxing hydrogels, such as hyaluronic acid networks, which are commonly crosslinked covalently, to have viscoelastic properties that more closely mimic tissue. The development and refinement of viscoelastic materials have added new complexity to relationships that had been well-established for elastic materials. For example, it had previously been shown that for mesenchymal stem cells (MSCs), adhesion to soft and stiff 2D elastic substrates biases differentiation towards adipogenic and osteogenic lineages, respectively [1, 17]. However, more recent studies with viscoelastic materials reveal that the rate of stress relaxation can strongly modulate stiffness-dependent differentiation, with rapid stress relaxation reducing adipogenic differentiation on soft materials and slow stress relaxation reducing osteogenic differentiation on stiff materials [18]. Such recent studies have also revealed how viscoelasticity effects mechanosensing [19, 20] and neurogenesis [21], and several excellent reviews cover the effect of viscoelastic cues on broad range of cell behaviors [6, 22–25]. Efforts to understand the basis of these striking results have yielded new mechanistic insights into how cells process viscous cues, which we discuss in depth later in the context of cell migration.

## 2. Measuring viscoelastic properties

We begin with a very brief overview of common rheological measurements taken for viscoelastic materials used for cell engineering, such as hydrogels for in vitro models. We refer readers interested a more extensive discussion to excellent past reviews on the subject [17, 26]. The rheology of a material describes the relationship between the stress (force/area) applied to the material and the resulting strain (fractional deformation). To a first approximation, the internal structure of a material can respond to stress in two ways: by absorbing or storing the stress through reversible deformation (elastic character) or by dissipating the stress through internal frictional interactions (viscous character). For example, a rubber band is an elastic material that stores applied stresses through molecular deformation within the constituent crosslinked polymer network. When the stretch is removed, the strain disappears, and the rubber band recovers its original shape. By contrast, water is an example of a purely viscous material, which dissipates applied shear stresses through internal flow, with friction between adjacent fluid layers releasing heat. In a purely and linearly elastic material, the stress is proportional to strain, whereas in a purely and linearly viscous (Newtonian) material, stress is proportional to the rate (first time derivative) of the strain. Many if not most biological materials are viscoelastic and can both store and dissipate applied stress.

**Figure 1.**
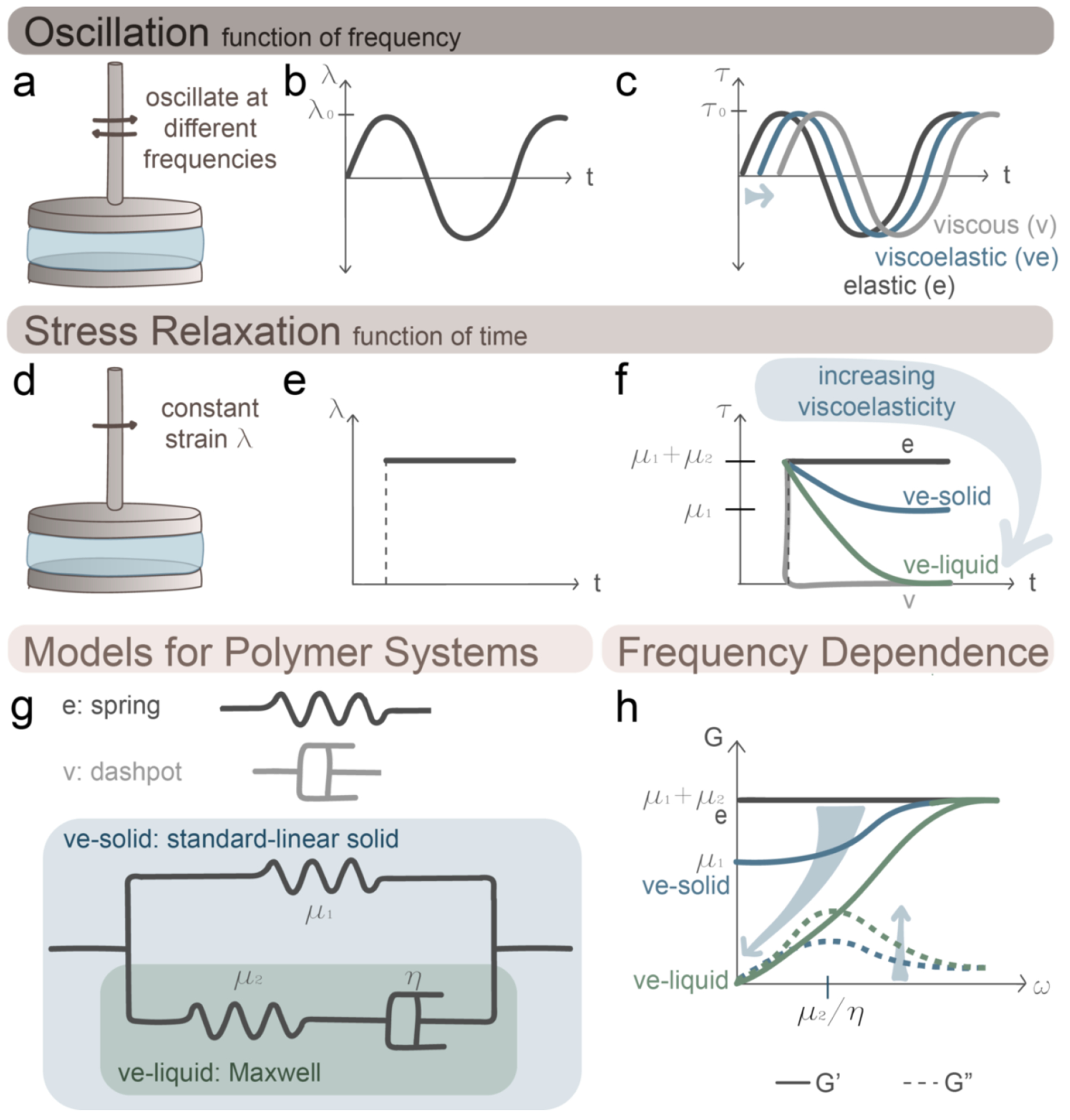
Shear Rheology for Polymer Systems. **(a)** In an oscillatory rheological measurement, the rheometer probe rotates sinusoidally at a given frequency (or over a “sweep” of frequencies, see (h) below), and thus **(b)** strain (λ) and **(c)** stress (⊤) oscillate with time (t). In a purely elastic material, ⊤ and λ are instantaneously coupled, whereas in a purely viscous material the two quantities are offset by a phase angle (δ)=π/2 (see text). Materials between these two extremes are considered viscoelastic. Unlike oscillatory tests, **(d)** stress-relaxation tests are performed at constant strain, so **(e)** strain and the resulting **(f)** stress change monotonically with time. A purely elastic material, such as an elastic solid, resists with a constant stress, reflecting storage rather than dissipation of the mechanical input. For a viscoelastic solid, stress falls (relaxes) with time to some plateau value, where the initial and plateau stress correspond to effective spring constants for the material ($\mu_{1}+\mu_{2}$ and $\mu_{1}$, respectively). For a viscoelastic fluid, such as a non-crosslinked polymer solution, stress eventually falls to zero. A purely viscous material does not hold stress at all. **(g)** All of these materials can be abstracted into models of springs and dashpots, where a purely elastic and purely viscous materials comprise of simply a spring or dashpot, respectively. Viscoelastic solids follow the standard-linear solid model, comprised of a spring in parallel with a spring and dashpot in series. Viscoelastic liquids, typically follow the Maxwell (spring and dashpot in series) model. **(h)** Each type of material responds characteristically when frequency is systematically varied (“frequency sweep”). Here storage (elastic) and viscous (loss) moduli are represented as G’ and G” respectively (see text), and the blue arrows represent progression from elastic to viscous character. G’ (solid lines) is constant for a purely elastic solid and increases with frequency for a viscoelastic material, as expected from (f) given that frequency represents a sort of inverse time. For a viscoelastic solid, $G^{’}=\mu_{1}$ at zero frequency and increases to a plateau value of $G^{’}=\mu_{1}+\mu_{2}$ at high frequency. For viscoelastic liquid, $G^{’}$ decays to 0 at the 0 limit. For G”, systems with a spring and dashpot in series have a maximum at $\mu_{2}/\eta$. Schematics are adapted from figures and text in the following references [27–29].

Viscoelastic properties are commonly measured with shear rheology, in which a sample of the material is placed between two surfaces, commonly a cone and plate or two parallel plates capable of rotation. A shear stress (or strain) is applied to one surface, and the resulting strain (or stress) is measured. In practice, rheological measurements are often obtained in an oscillatory (“AC”) fashion, which enables high measurement sensitivity at comparatively small stresses/strains by taking advantage of relationships between trigonometric functions. In this scenario, stress (τ) and strain (γ) are applied and/or measured as time (t)-dependent wavefunctions with some frequency (ω) (Fig 1a–c). For example, a purely linear elastic material experiencing a sinusoidal time-dependent strain $\gamma =\gamma_{0}sin(\omega t)$, will exhibit an instantaneous, sinusoidal stress $\tau =\tau_{0}sin(\omega t)$, because stress is proportional to strain ($\tau_{0}$ and $\gamma_{0}$ are the stress and strain amplitudes) (Fig 1c). Conversely, a purely and linearly viscous material subjected to a time-dependent sinusoidal strain will exhibit a delayed time-dependent stress in the form of a cosine ($\tau =\tau_{0}cos(\omega t)$), because stress is proportional to the rate of strain, and a cosine is the first derivative of a sine (Fig 1c). The degree to which the time-dependent stress curve is offset from the time-dependent strain curve is captured in the phase lag or phase angle (δ, where $\gamma =Y_{0}sin(\omega t)$ and $\left.\tau =\tau_{0}sin(\omega t+\delta )\right)$. Since the radial offset between a sine and cosine is π/2, the value of δ lies within in the interval 0≤δ≤π/2, serving as a convenient measure of viscoelastic character. In this framework, δ=0 for purely elastic materials and δ=π/2 for purely viscous materials. Therefore, for frequency-dependent shear moduli, δ can be combined with the complex modulus ($G^{*}$, the ratio of the maximal stress to the maximal strain) to obtain the elastic or storage (G’) and viscous or loss (G”) moduli: $G’=G^{*}\cos(\delta )$ and $G”=G^{*}\sin(\delta )$, with $G^{*}=G^{’}$ for purely elastic materials and $G^{*}=G^{”}$ for purely viscous materials [26, 27].

The time-dependent mechanics of viscoelastic materials have two important implications that recur throughout our discussion. First, viscoelastic materials do not dissipate stress instantaneously but do so in a time-dependent fashion, with more stress dissipated the longer the stress is maintained. This phenomenon can be measured in a stress-relaxation (constant strain) (Fig 1d–f) or creep-response (constant stress) measurement. Whereas a viscoelastic solid eventually resists and stores the applied strain and the measured stress plateaus, a viscoelastic liquid continuously dissipates the applied strain, and the measured stress falls to zero (Fig 1f). Like the phase angle δ, stress dissipation serves as a convenient measure for viscoelastic character, where the faster or slower a material can dissipate stress, the more viscous or elastic it is, respectively (Fig 1f). The time to reach half the initial stress ($T_{1/2}$) serves as a quantitative measure, where faster stress relaxation is accompanied by a smaller $T_{1/2}$ value. We note that much deeper and more rigorous descriptions of terms used to describe viscoelastic materials may be found elsewhere [6, 22, 23]. Second, viscoelastic properties are highly frequency-dependent, with both G’ and G” rising with the frequency of the stress/strain. Viscoelastic solids, such as polymer hydrogels, often follow the standard-linear solid model, where a spring (frequency-independent elastic shear stiffness $\mu_{1}$) is in parallel with a dashpot (viscous unit η) and spring (viscoelastic shear stiffness $\mu_{2}$) in series, whereas a viscoelastic liquid, such as an entangled but non-crosslinked polymer solution, follows the Maxwell model ($\mu_{2}$ in series with η) (Fig 1g). At high frequencies, G’ is independent of frequency for both elastic and viscoelastic solids, plateauing at the value $G^{’}=\mu_{1}+\mu_{2}$, because the stress is delivered more rapidly than the material can dissipate it (Fig 1h). While elastic solids do not have the ability to dissipate stress (G’ remains constant at this value), viscoelastic solids can start to dissipate stress below a characteristic infinite-frequency limit (“plateau frequency”), with G’ falling with lower frequencies (longer periods of time). However, as frequency approaches the zero limit, G’ will have another plateau ($G^{’}=\mu_{1}$), corresponding with the stress-relaxation plateau value at infinitely long timescales, where the network lacks additional capacity to dissipate stress (Fig 1h). In practice, it is often easier and more accurate to measure this value in a static stress-relaxation experiment vs. an oscillatory measurement, given that measurement times can typically be extended much more accurately and precisely than frequencies can be shortened. Lastly, G” does not necessarily always increase with frequency; instead, in the cases of both viscoelastic solids and liquids, where $\mu_{2}$ is in series with η, G” has a maximum at $\mu_{2}/\eta$ [27–29] (Fig 1h).

## 3. Types of materials

Here we will discuss the viscoelastic properties (and efforts to engineer these properties) of four common biomaterials: collagen I, alginate, hyaluronic acid, and electrospun fibers. We have limited our focus to these materials because of their tunability and widespread use in basic and translational applications. Nonetheless, many of the themes discussed below can be used to approach other materials, many of which are explored in a recent review [24].

### 3.1 Collagen I

**Figure 2.**
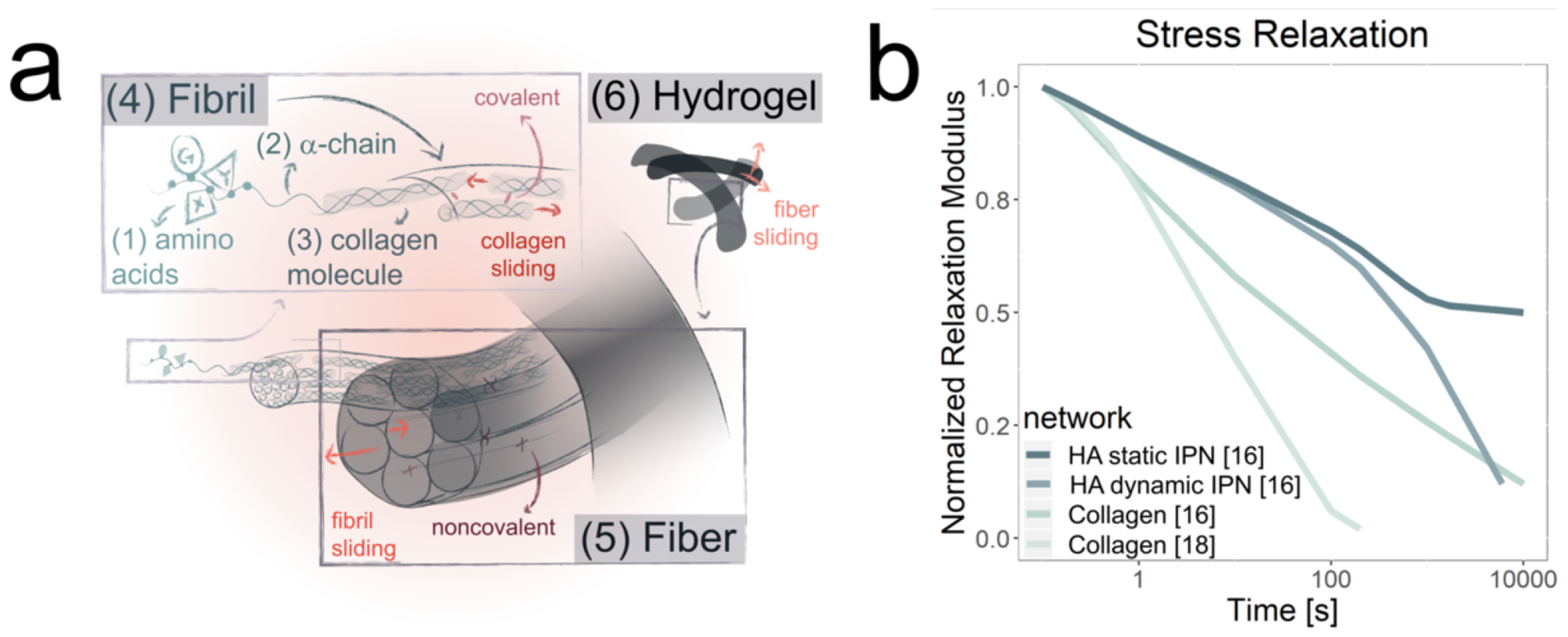
Architecture and stress-relaxation relationships for Collagen. **(a)** Schematic showing the multiscale architecture of collagen, along with how properties of the network lead to viscoelastic properties. Collagen hydrogels are characterized by a hierarchical, fibrillar structure encoded within the primary sequences of the protein chains. Collagen hydrogels derive viscoelastic properties from a combination of entanglement, crosslinking (covalent and noncovalent), and fibril/fiber deformation and sliding. (b) Experimental stress-relaxation curves compiled and reformatted from two different studies [16, 18]. Collagen hydrogels [16, 18] relax stress faster than HA-collagen IPNs [16].

The extracellular matrix (ECM) protein collagen makes up ~30% of total body protein mass and plays essential roles in tissue structure and mechanics. While 28 types of collagen have been described, ~90% of the body’s collagen consists of the fiber-forming collagen I. Collagen I is most abundant in connective tissues, such as tendons, skin, bone, and lungs, giving these tissues mechanical strength and elasticity. This fibrous network can be broken down in a hierarchical fashion to highlight the origins of its viscoelastic nature (Fig 2a). The basic building block of collagen I is an alpha chain encoded by the COL1 gene, which like all collagens, self-assemble into triple helical structures (1.5 nm diameter) mediated by glycine-X-Y repeats [30, 31]. Here, X and Y are predominantly proline and hydroxyproline, the latter of which is formed from posttranslational modification of proline residues [30, 31]. The triple helices then self-assemble by staggering in parallel into a collagen fibril (20-280 nm diameter), with hydroxypolines and hydroxylysines frequently participating in inter- and intramolecular covalent crosslinks [30, 31]. Vitamin C (ascorbate) is an essential cofactor in hydroxyproline and hyroxylysine synthesis, which accounts for the catastrophic loss of tissue integrity seen in chronic vitamin C deficiency (scurvy). The fibrils then bundle together to form thicker collagen fibers (1-300 μm), where proteogylcans such as decorin can noncovalently crosslink the fibrils together to increase strength and stability [30–37]. In vitro, these large collagen fibers can entangle with each other to form a hydrogel with complex viscoelastic properties. In tissue, collagen I is often highly ordered, with fibers organizing into specialized architectures, such as parallel bundles (tendon, ligament), orthogonal lattices (cornea), concentric rings (bone, blood vessels), and basket weaves (skin) [38, 39]. The relationship between these tightly regulated in vivo geometries and mechanical properties remains incompletely understood and is an area of active study.

Due to its high relevance to connective tissue ECM and abundant availability from animal sources, collagen I (hereafter simply referred to as collagen) is perhaps the most frequently used natural biomaterial in the laboratory, clinic, and marketplace. When reconstituted in the laboratory, collagen can form a three-dimensional (3D) hydrogel stabilized by fiber branching and entanglement. Of high practical importance, collagen hydrogel assembly can be triggered from solution through a step change in temperature or pH. While this may appear to function as a simple “switch,” the mechanism of collagen hydrogel assembly is very complex, with hydrogel structure and mechanics heavily influenced by kinetic factors that can be difficult to control, such as the rate of temperature change [40]. Moreover, because collagen is typically purified from tissue rather than recombinantly expressed, there can be significant variation within and between tissue sources [41]. Reconstituted collagen hydrogels also differ from tissue collagen in at least two important respects. First, spontaneously assembled collagen gels lack the ordered architecture of tissue collagen, which as noted above, is often key to structural and mechanical functions [38, 42] and can give rise to nonlinear elastic properties (e.g. strain-stiffening) [43]. Second, collagen fibers in tissue are frequently decorated with accessory factors, such as fiber-associated collagens and proteogylcans, which both assist in crosslinking and tightly regulate fiber length, diameter, and organization [32]. The specific form of crosslinking significantly influences both the mechanical properties of collagen scaffolds and how cells integrate into these scaffolds [44, 45]. Without the crosslinking mechanisms normally found in tissue, reconstituted collagen matrices typically lack the high elasticity and tensile strength of collagen-rich tissues. For the same reason, collagen hydrogel mechanics depend strongly both on tissue source and extraction method, particularly the extent to which native crosslinks are retained, and these differences may strongly influence cell responses.

Because collagen self-assembly is driven primarily by physical entanglement rather than covalent crosslinking, collagen hydrogels are inherently viscoelastic due to dissipation of imposed stresses by fiber slipping and rearrangement. While much of the field’s mechanistic understanding of collagen viscoelasticity is based on inferences from bulk measurements, increasing effort has been made to bridge length scales by explicitly connecting bulk and microscopic measurements [33, 37, 43, 46–49]. For example, computational creep tests performed on collagen-like peptides treated as Kelvin-Voight (KV) elements have revealed strong nonlinear viscoelastic behavior, with molecular-scale viscosity orders of magnitude lower than past measurements of fibrils [33]. Scaleup of the model by constructing grids of KV elements in series and parallel captured some but not all fibril properties, suggesting that other mechanisms contribute to fibril mechanics, such shearing and displacement of water between the triple helices [33, 34, 50]. Additional studies further support the idea that the relative mobility of fibers and fibrils within the network enables further stress dissipation [51–54]. For example, one set of experimental stress-relaxation measurements of collagen gels revealed a range of characteristic relaxation times spanning <1 s to >100s, which the authors attributed to fiber/fibril sliding at different length scales [37]. On the macroscopic scale, the viscoelastic behavior of collagen tissues and hydrogels has been widely explored [37, 43, 46, 47, 55–69].

The tools of materials engineering have proven valuable for the manipulation of collagen stress-relaxation capabilities. This is especially useful when trying to mimic tissue, for the absence of noncovalent crosslinking proteins in reconstituted collagen hydrogels leads these materials to relax stress faster than collagen-rich tissue and other tissue-based biomaterials [18]. One approach to decrease collagen’s stress-relaxation ability is to create an interpenetrating network (IPN) with another, less stress-relaxing material. For example, formation of an IPN between collagen and hyaluronic acid (HA) formed through both supramolecular and covalent crosslinking reduces the stress-relaxation abilities below even that of native tissue [16, 18] (Fig 2b). Other approaches achieve a similar effect by introducing tissue-like or tissue-derived crosslinkers, by suspending the collagen hydrogel in a proteoglycan matrix, or by using chemical crosslinking reagents such as genipin, glutaraldehyde, EDC-NHS, procyanidin, or photo-active compounds [33, 37, 43–45, 49, 70–72].

### 3.2 Alginate

**Figure 3.**
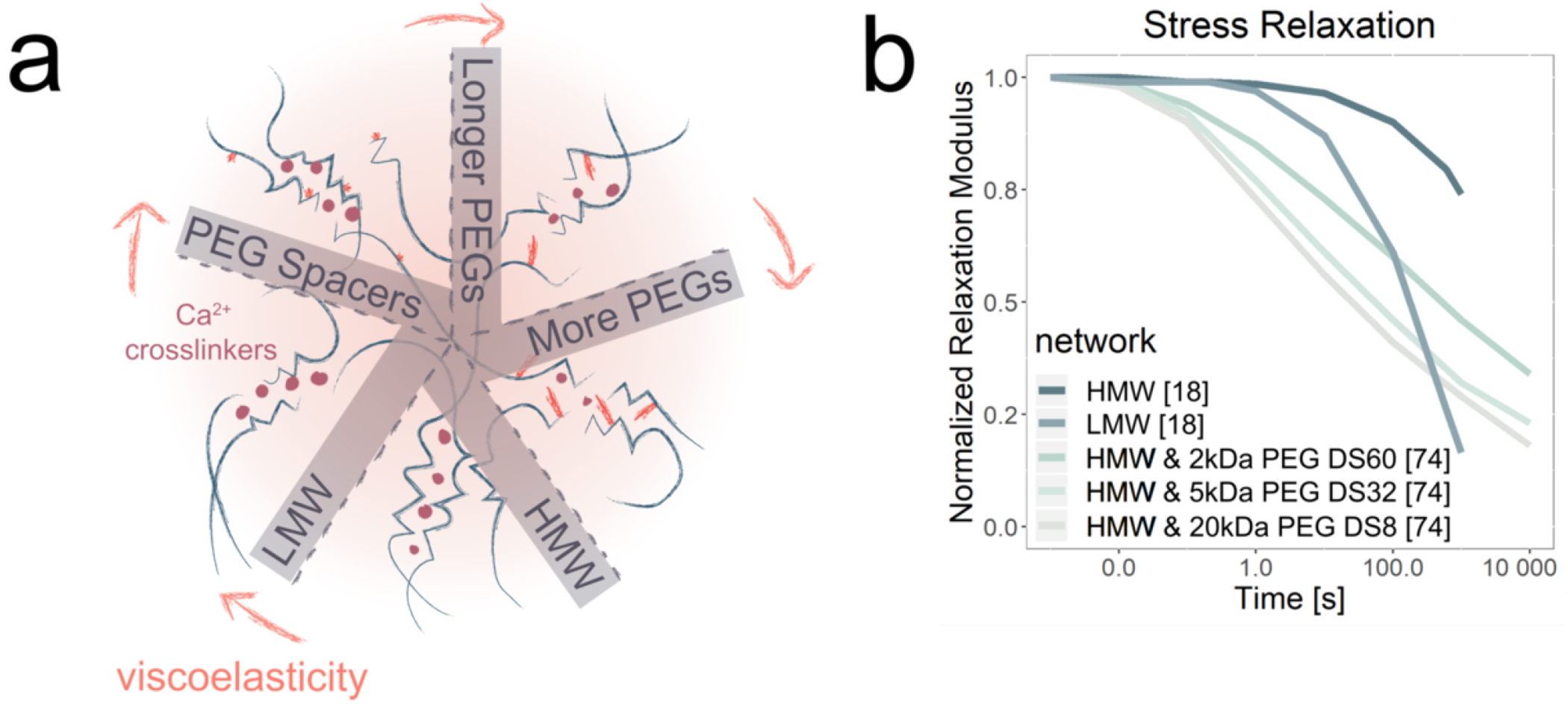
Architecture and stress-relaxation relationships for Alginate. **(a)** Schematic of alginate network architecture, showing how the network may be modified to tune viscoelastic properties by controlling alginate molecular weight and by introducing PEG spacers between the alginate chains. **(b)** Experimental stress-relaxation data compiled and reformatted from two different studies [18, 74]. Decreasing alginate MW and increasing PEG spacer MW tends to enhance viscous character. Further, a smaller degree of saturation (DS) is needed for a larger PEG spacer [74].

Another common and inherently viscoelastic biomaterial is alginate, a plant-derived polysaccharide that can be noncovalently assembled into hydrogels through calcium ion-mediated crosslinking. Alginate has emerged as an attractive ECM biomaterial due to the ease with which it can be reconstituted and chemically modified. As with collagen, alginate is naturally viscoelastic due to the ability of the network to dissipate stresses by breaking and reforming noncovalent crosslinks. Alginate’s viscoelastic properties can be further tuned to achieve targeted stress-relaxation kinetics (Fig 3a). When the molecular weight (MW) of the alginate chains decreases, the chains rearrange more easily, leading to faster stress relaxation [73] (Fig 3b). Hydrogel stress relaxation may be further enhanced by appending PEG spacers to the alginate chains, which sterically hinders crosslinking, leading to further stress relaxation (Fig 3b). As expected, this steric hinderance not only depends on the MW, but also the density of the PEG spacers, where one parameter may be tuned to compensate for changes in the other. For example, equivalent stress-relaxation rates may be achieved either by using a low density of HMW PEG spacers or a high density of LMW PEG spacers [18, 74] (Fig 3b).

The mechanical properties of alginate hydrogels may be further tuned by incorporating covalent crosslinking with a diamine crosslinker, such as adipic acid dihydrazide (AAD), which reacts with alginate’s carboxylic acid. By using covalent crosslinking, the network cannot break and reform bonds, rendering the network more elastic with slower stress-relaxation capabilities [75, 76]. With increasing amount of crosslinker, covalently [75] and ionically [76] crosslinked alginate relax stress more quickly.

### 3.3 Hyaluronic Acid

Hyaluronic acid (HA), an anionic, non-sulfated glycosaminoglycan, is another natural biomaterial that is abundant in a variety of tissues. While HA is classically associated with the ECM of brain and cartilage, it is also found in skin, muscle, and other connective tissues. HA-dominant ECMs such as brain ECM lack the fibrillar architecture of collagen-based ECMs and instead consist of a dense meshwork in which HA is noncovalently organized by large proteins (tenascin and lectins) [8]. Together with the high water content driven by HA’s extreme hydrophilicity, the transient binding and unbinding of these crosslinks contributes to the viscoelastic properties of brain tissue.

When HA hydrogels are reconstituted in the laboratory, HA is typically either co-gelled with a second network (e.g. collagen) or covalently modified to introduce chemical “handles” that permit covalent crosslinking. Commonly introduced moieties include acrylates, methacrylates, thiols, norbornenes, and cyclic alkynes, which can then be crosslinked with a variety of efficient chemistries, often in the presence of cells. For example, our own work has made extensive use of HA-methacrylate crosslinked with dithiols [77, 78] or LAP photo-initiators [79] and HA-dibenzylcyclooctyne (DBCO) crosslinked with diazides [80].

**Figure 4.**
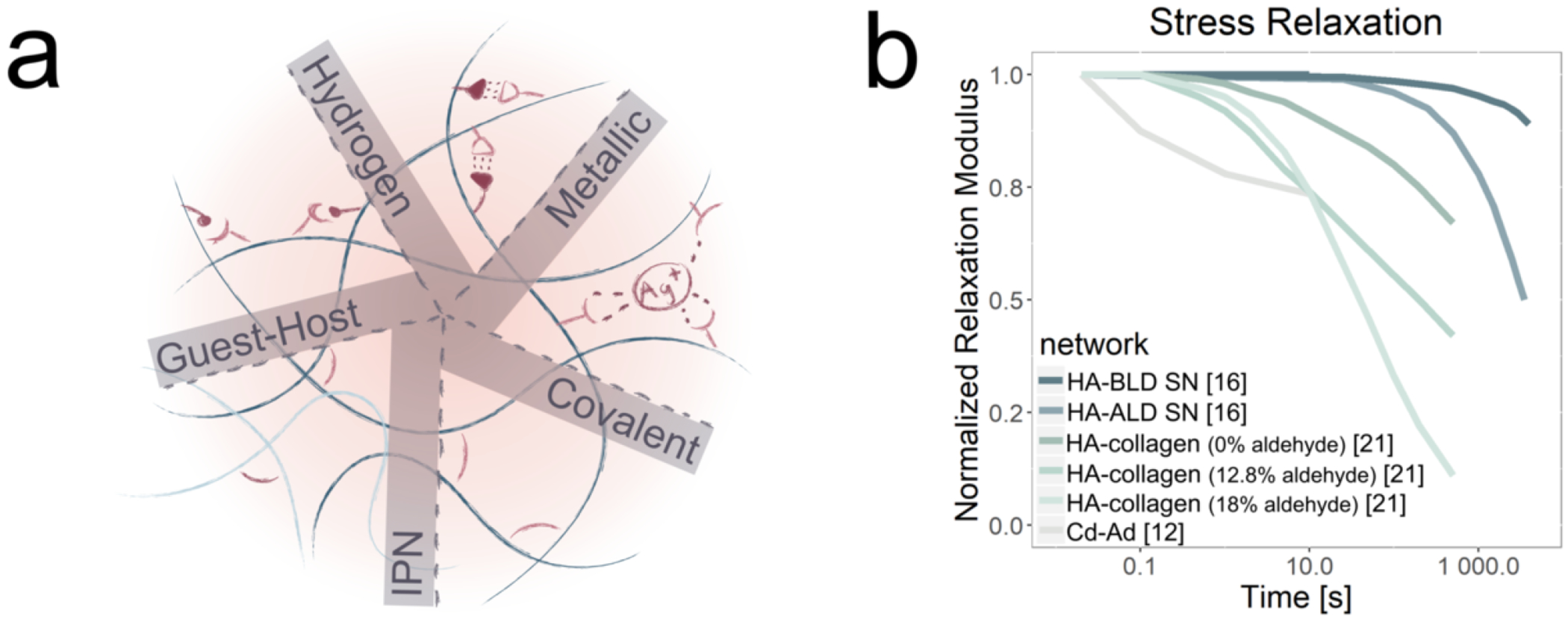
Architecture and stress-relaxation relationships for Hyaluronic Acid (HA). **(a)** Schematic of HA network architecture of HA, showing how HA networks may be modified to enhance viscoelastic properties. HA hydrogels may be transiently crosslinked to introduce viscoelastic character via IPNs, hydrogen bonds, coordinated metal ions, and host-guest chemistries. **(b)** Experimental stress-relaxation data compiled and reformatted from three different studies. Cd-Ad-based crosslinking [12] and IPN networks of HA-collagen [21] stress relaxes faster than hydrazone crosslinking (HA-ALD and HA-BLD single networks) [16].

While it has been challenging to precisely match stress-relaxation time scales seen in tissue HA matrices, the use of crosslinks that can be broken and reformed under force, such as cyclodextrin-adamantane (Cd-Ad) host-guest chemistries and hydrazine-aldehyde dynamic covalent bonds (hydrazone bond), allows introduction of more pronounced viscoelastic properties (Fig 4a). The structure and chemistries of these supramolecular crosslinkers play an important role in the kinetics of breaking and reforming bonds, and thus, stress dissipation. With respect to the hydrazone bond, an aliphatic aldehyde (HA-ALD single network (SN)) can stress relax much faster than the benzyl aldehyde (HA-BLD SN) due to less steric hinderance [16] (Fig 4b). Further, a higher HA MW dissipates stress even slower, while the addition of a collagen IPN enables faster stress dissipation. Recent studies have also used dynamic bonds to connect HA to other stress-relaxing materials, such as an imine-aldehyde bond to collagen [21] or Diels-Alder bond to Chondroitin Sulfate (CS) [81]. The HA-collagen network can be further manipulated to have even greater stress relaxation by increasing the percentage of aldehyde from 0 to 18% (Fig 4b). However, the Cd-Ad hydrogel can stress relax several orders of magnitude faster than the HA-ALD and HA-collagen hydrogels, but plateaus at a higher $\mu_{1}$ value due to its non-dissipative covalent crosslinks [12, 16, 21] (Fig 4b). Other host-guest chemistries such as cyclodextrinazobenzene (Cd-Azo) and cucurbit[6]uril-polyamines (CD[6]-PA) have been used for biomedical applications, but their viscoelastic properties have not been thoroughly explored [82]. The molecular structure of Cd is cone-shaped and CB[6] is ball-shaped, which may contribute to differences in affinity and stress-relaxation kinetics of the two crosslinking chemistries [83]. A recent review provides an extensive analysis of the kinetics of reversible bonds [84], and there have been several additional rheological studies of host families Cd [9–14] and CB[6] [15].

A variety of other noncovalent HA crosslinking strategies have been explored and reviewed elsewhere [85]. For example, HA has been crosslinked by appending nitrogenous bases, such as cytosine and guanosine, which hybridize via hydrogen bonds [86] (Fig 4a). Electrostatically crosslinked HA networks have also been formed using positively-charged crosslinkers, such as multivalent poly(2-aminoethyl methacrylate) (PAEM) [87] or unfolded Bovine serum albumin (BSA) [88]. HA may also be decorated with moieties that can chelate and coordinate metal cations [89–96] (Fig 4a). These hydrogels have been used for injection and self-healing applications, but the ways in which their chemical compositions can be manipulated to tune viscoelastic properties remain an open area of study.

### 3.4 Electrospun Fibers

**Figure 5.**
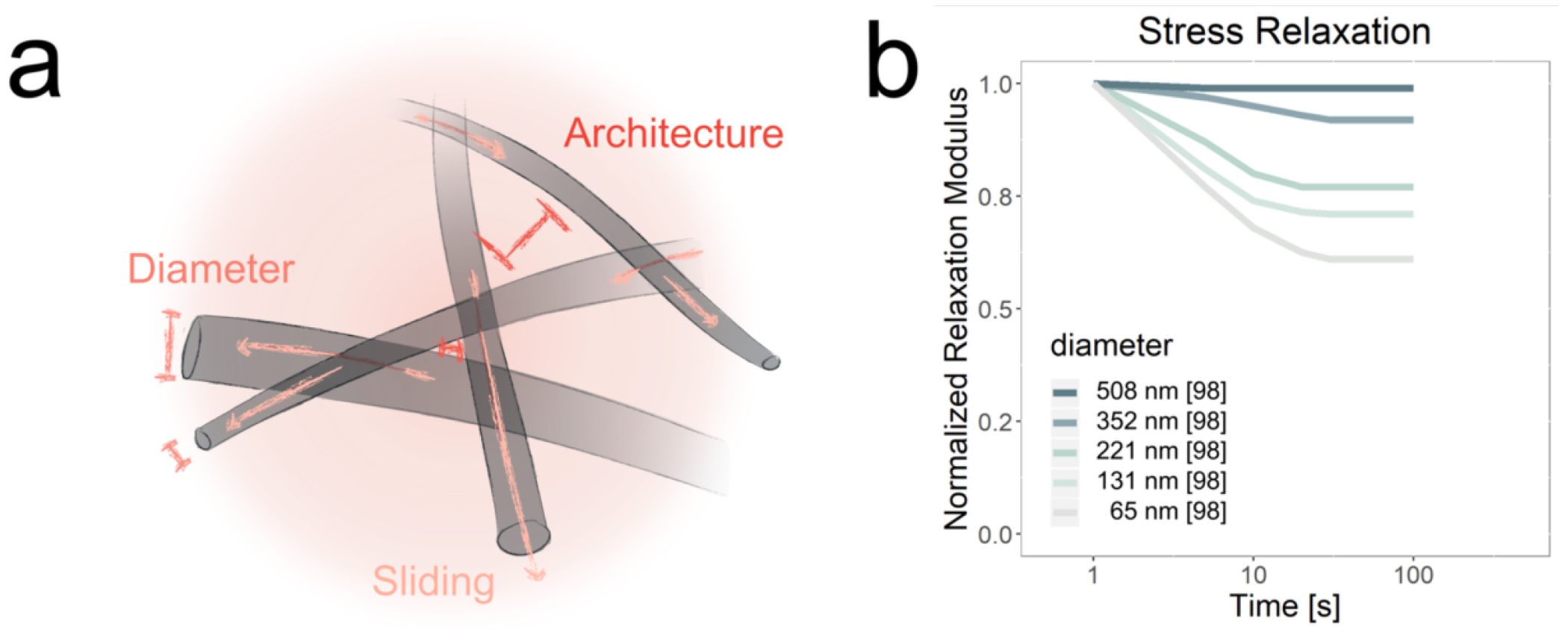
Architecture and stress-relaxation relationships for Electrospun Fibers. **(a)** Schematic showing network architecture of electrospun fibers, along with how this network facilitates viscoelastic properties. Electrospun fibers derive viscoelastic properties analogously to collagen, due to their common fibrillar architecture, where fiber diameter, sliding, and density all play a role. **(b)** Experimental stress-relaxation curves compiled and reformatted from a single study [98]. As fiber diameter increases, the extent of stress dissipation also increases.

As noted above, natural biomaterials such as collagen derive viscoelastic properties from their fibrous architecture. Fibrous architecture can be introduced into synthetic biomaterials by deploying these materials as electrospun fibers. While many methods have been developed to fabricate these micro- and nano-scale materials, perhaps the most common approach is to extrude a polymer solution in the presence of an electric field, where repulsion between like charges on the droplet surface deforms the droplets into threads that can be solidified and collected. Electrospun nanofiber fabrication has proven highly attractive due to its compatibility across a wide variety of chemical compositions and the ease with which fiber properties (e.g. diameter) may be engineered by controlling elecrospinning conditions [97]. Electrospun fiber-based materials are expected to exhibit viscoelastic properties through the same mechanisms as natural fiber-based materials, i.e. dissipation of stress through fiber slippage and rearrangement. These mechanisms are explained in further detail in the collagen section. That said, is important to note that the viscoelastic properties of electrospun networks remain very incompletely characterized. Because the features of electrospun fibers (and the resulting networks) can be engineered much more precisely than natural fibrous materials, they offer a prime opportunity to dissect relationships between structure and mechanics. For example, one study has demonstrated that electrospun fiber network stress relaxation may be engineered by controlling the diameter of the constituent fibers [98] (Fig 5b). Stress-relaxation measurements of electrospun PVA from this and a related study reveal that stresses plateau and do not dissipate to zero [98, 99]. Adding motifs, such as the host-guest chemistries of Cd-Ad [100] or dynamically covalent hydrazone bonds [101], introduces additional properties, such as shear-thinning and self-healing, and perhaps most pertinently to viscoeastic properties, rearrangement under mechanical stress. A relatively new fabrication strategy based on fragmenting and reassembling electrospun fibers offers an additional degree of tunability [102, 103]. Much additional opportunity remains to characterize effects of fiber alignment and density on viscoelasticity.

There is ripe opportunity to engineer electrospun fiber networks that capture defining features of natural fibrous materials through control of inter-fiber entanglement and mobility, which would presumably influence stress dissipation. The use of electrospun fibers as well-controlled mimics of native ECM scaffolds has already yielded great insight into mechanisms through which cells sense mechanical and topographical cues in 3D matrices [104–107].

### 3.5 Summary

We can draw comparisons between the origin and manipulation of these materials’ viscoelastic properties. The fibrous character of collagen and electrospun materials allows each network to dissipate stress through fiber slippage and rearrangement. Similarly, alginate is also commonly crosslinked noncovalently, allowing it to stress relax through association and dissociation of the crosslinks, whereas hyaluronic acid is most commonly crosslinked covalently, inhibiting fast stress relaxation. That said, crosslinking strategies traditionally associated with one material can be used for other materials. For example, collagen and alginate may be covalently crosslinked with genipin and diamine crosslinkers, respectively, and HA may be dynamically crosslinked, e.g. using adamantane/cyclodextrin host-guest interactions.

## 4. Cell Motility

We now discuss how scaffold viscoelastic properties influence migration in two-dimensional and three-dimensional matrices.

### 4.1 Two-dimensional motility

**Figure 6:**
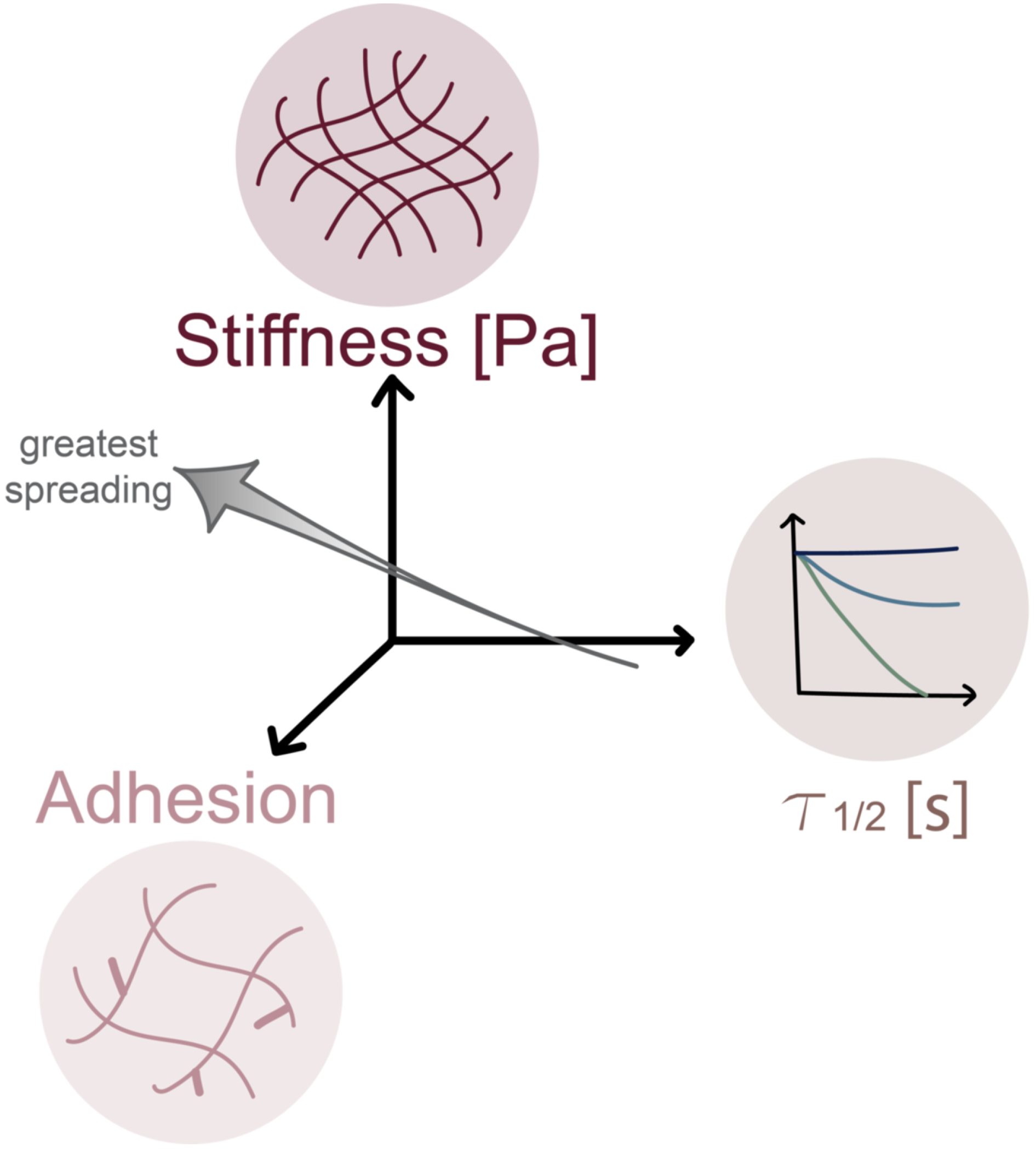
2D Cell Spreading. A general theme from 2D motility studies is that when a sufficiently high density of adhesive ligands is provided on stiff, fast-relaxing hydrogels, cells spread more on viscoelastic surfaces than on their elastic counterparts.

The vast majority of cell biological studies continue to be performed in 2D culture due to its ease of setup, high throughput and parallelizability, and strong compatibility with optical imaging. On purely elastic materials, cell spreading depends strongly on substrate elasticity and adhesivity, with spread area commonly observed to increase with both parameters up to some maximum [77, 108–112]. These substrate-dependent increases in area are typically accompanied by greater assembly and/or area of integrin-dependent adhesive complexes. Substrate viscoelastic properties and adhesivity may cooperatively regulate spreading in unexpected ways. For example, at low adhesivity (e.g. RGD peptide concentration), fibroblasts have been reported to spread more robustly on elastic, covalently-crosslinked alginate hydrogels than on ionically crosslinked, stress-relaxing alginate hydrogels [75]. When adhesivity is increased, cells spread more on stress-relaxing hydrogels than on elastic hydrogels and produce stress fibers. On all four modulations of adhesive and viscoelastic character, cells increase their spreading area as stiffness increases. Revisiting the materials discussed earlier in which dynamic HA-collagen IPNs are crosslinked with hydrazone-based chemistry, MSCs on HA-ALD IPN gels spread in a manner that was grossly independent of stress-relaxation time. Cells on HA-BLD IPN gels were rounder on slower-relaxing gels and spread more on gels with faster stress relaxation [16]. The opposite trend was observed for HA Cd-Ad gels: RGD-decorated, elastic hydrogels supported greater cell spreading than on their viscoelastic counterparts, where cells on both surfaces also increase spreading as stiffness increases [14]. This discrepancy may be a function of the differences in stress relaxation of Cd-Ad and hydrazone crosslinked hydrogels, where the Cd-Ad gel stress relaxes quickly but plateaus at a higher $\mu_{1}$ value due to its non-dissipative covalent crosslinks. In addition to the density of adhesive ligands, the type of adhesive moiety matters as well; for example, a fibronectin fragment Fn9*10 supported less spreading than RGD on the Cd-Ad gels [13]. Similar to the Cd-Ad gels, greater stress relaxation is achieved on the elastic compared to viscoelastic polyacrylamide gels, which could also be attributed to covalent crosslinking, lack of plasticity within the network, how protein is affixed to the gel, and differences in cell and matrix timescales in the motor-clutch model (explained in the next paragraph) [113]. Thus, a general theme (but not absolute rule) from these studies is that when a sufficiently high density of adhesive ligands is provided on stiff, fast-relaxing hydrogels (smaller $\tau_{½}$), cells spread more on viscoelastic surfaces than on their elastic counterparts (Fig 6).

For purely elastic surfaces, cell migration speed is classically observed to depend biphasically on substrate stiffness [108, 114]. On very soft surfaces, cells are unable to generate sufficient contractile force to support tension-dependent focal adhesion maturation, preventing migration. On very stiff substrates, high tension-dependent reinforcement of adhesions suppresses the turnover of adhesions needed for productive motility. Intermediate-stiffness surfaces balance adhesion formation and turnover and thus support the fastest migration. This relationship has been framed in terms of a motor-clutch model in which cellular process extension is driven by a relationship between protrusion, myosin-driven retrograde flow, and the molecular “clutches” that couple the two processes [115, 116]. These relationships change on viscoelastic substrates, due to introduction of a relaxation timescale that competes with other timescales within the model, including clutch binding [117, 118]. The viscoelastic motor-clutch model describes that elevated stiffnesses saturate the binding clutches, leading to similar spreading areas, which is also the outcome when substrate relaxation timescale is much faster than the clutch binding timescale [117]. This relationship between the hydrogel mechanics and clutch engagement may explain the differences in cell spreading between the hydrogels described above. A new study found that the intermediate filament vimentin also regulates cell adhesion and spreading on viscoelastic substrates [119].

Another study reveals cells migrating minimally on soft, elastic hydrogels, but much more robustly on soft stress-relaxing hydrogels [120]. In addition to changing migration speed, introduction of viscous cues can also alter the mode of migration. Cells have been observed to shift from a lamellipodial mode of motility on stiff, elastic matrices to filopodia-based migration involving fewer, weaker adhesions on viscoelastic surfaces [120]. Analogous efforts have been made to understand how collective migration changes between elastic and viscoelastic surfaces [121–123].

### 4.2 Three-dimensional motility

The 3D geometry of tissue figures centrally in the progression of many processes in physiology and disease, such as organoid formation and cancer invasion. Modes and mechanisms of 3D migration have been the subject of extensive study, but the field’s understanding of how viscoelasticity regulates 3D motility is still emerging [124]. In 3D, the role of viscoelasticity becomes even more complex because it influences and is influenced by many other factors known to regulate 3D migration speed, including pore size, degradability, and viscoplasticity [6]. Matrix viscoelastic properties may be leveraged by cells to facilitate motility in sterically confining environments. For example, in the absence of other adaptive mechanisms, cells are often unable to migrate effectively through 3D matrices with pore sizes below ~3 μm, which is typically the case for alginate and hyaluronic acid hydrogels [6, 125]. These steric barriers can be overcome through a combination of matrix degradation and deformation, which are coupled in interesting ways. For example, local matrix degradation also changes local viscoelastic properties. Degradation creates smaller, non-crosslinked polymer chains, increasing local viscous character, a phenomenon that has been demonstrated by microrheology [126].

**Figure 7:**
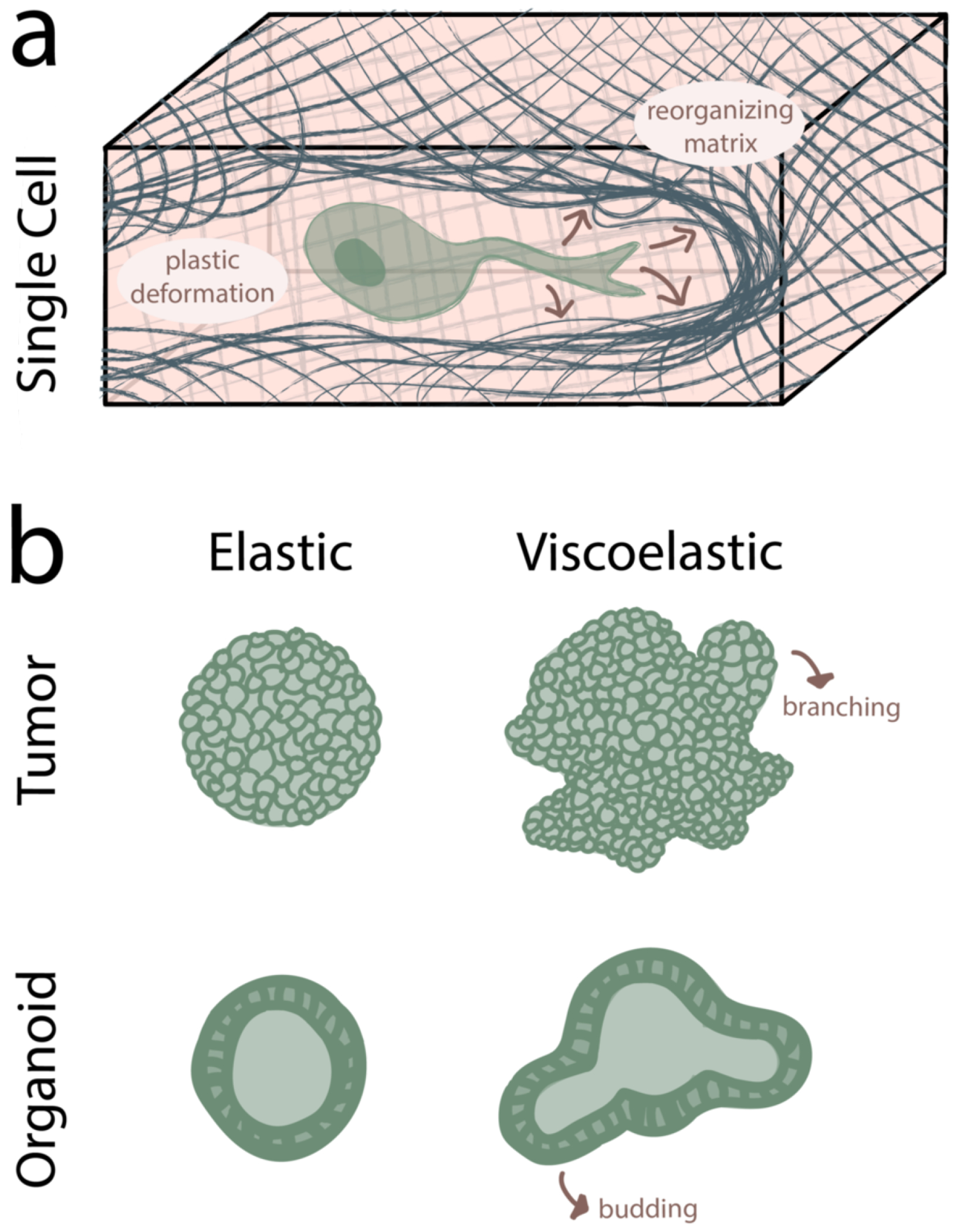
3D Cell Motility. **(a)** In 3D viscoelastic hydrogels, cells can viscoplastically deform and displace network chains to open cell-sized pores to facilitate migration. **(b)** This network rearrangement allows large groups of cells in tumors and organoids to facilitate branching and budding

When the matrix is not degradable, cells may exploit the mechanical plasticity of the matrix to overcome steric barriers to motility, with cells deforming and reorganizing the matrix through stretching and softening in order to creating a path for migration [124] (Fig 7a). In one study, cancer cells were observed to use invadopodia to force open migration channels in highly plastic alginate-rBM (reconstituted basement membrane) IPN hydrogels [125], whereas in another study the force for path creation was provided by a nuclear piston mechanism [73]. Recently, a chemo-mechanical model has been developed to investigate how pores are opened via a number of intracellular factors including myosin recruitment, actin polymerization, matrix deformation, and activation of mechanosensitive signaling pathways [127]. Cyclical actin polymerization and myosin recruitment produces invadopodia, whose progressive advance plastically deforms the matrix to open a migration channel. An important advance of this model is that it incorporates and independently manipulates plastic (permanent) matrix deformation in addition to viscoelastic properties. A discrete model has also been created to show how matrix properties, such as fiber concentration and amount of crosslinker, regulate matrix remodeling and stress profiles, which was validated with fibroblasts in a collagen matrix [128]. As predicted, higher fiber concentration and crosslinking leads to less remodeling. Many studies have also characterized the ways that cells mechanically remodel collagen networks via plastic deformation, such as strain-stiffening and fiber reorientation, with fiber reorientation of creating contact guidance cues for migration [64, 122, 129–132]. Not only were cancer cells predominantly found to be oriented on aligned collagen fibers in mammary tumors, but also once explanted onto randomly ordered collagen fibers, they radially aligned them to facilitate faster invasion [133]. Ion channels have also been proposed to drive 3D motility through an osmotic engine mechanism, the activation of which presumably would be regulated by matrix viscoelastic properties. A number of creative strategies are lending insight into mechanisms of path creation, such as the use of fluorescent microbeads to track matrix deformation during migration [73] and the development of new computational models of cell migration through materials of defined viscoelastic properties [134]. An important challenge for the field will be to determine the significance of regulatory events gleaned from reductionist 3D viscoelastic materials in complex physiologic and disease settings.

Similar to the general theme seen with 2D spreading, cells have also been observed to spread more in 3D matrices with high adhesivity, stiffness, and fast stress relaxation. For example, in rapidly stress-relaxing alginate hydrogels, MSCs elongate and proliferate more rapidly with increases in relaxation speed [18, 74]. Chondrocyte spreading and proliferation also increase with faster stress relaxation due to reduced confinement and osmotic pressure [135]. Further, in PEG hydrogels dynamically crosslinked with hydrazones, myoblasts spread more on viscoelastic aliphatic aldehyde (ALD) gels vs elastic-like benzaldehyde (BLD) gels [136]. Similarly, MSC volume increases in viscoelastic boronate-crosslinked PEG hydrogels [137]. Lastly, the importance of stress-relaxation amplitude was shown in a study of fibroblast migration speed, proliferation, and circularity in gelatin-alginate hydrogels [138].

Beyond the migration of individual cells, viscoelastic properties are increasingly understood to control collective cell migration, such as in morphogenesis and tumor invasion [139]. Recent work shows that in viscoelastic alginate matrices, MCF10A spheroids and intestinal organoids not only increase in cross-sectional area, but also decrease circularity either through branch formation or budding (Fib 7b). Cells within the branches also display high FAK phosporylation, nuclear YAP, and proliferation. Differences seen in spheroid area and circularity become more drastic as stiffness increases. Small intestine organoids cultured in reversible hydrogen bonded PEG hydrogels showed greater budding in materials with greater viscoelastic character [140]. As yet another example, human induced pluripotent stem cells undergo apoptosis with reduced matrix adhesivity and stress relaxation, whereas an increase in both of these parameters leads to lumen formation [141].

### 4.3. Summary

While the field’s understanding of how viscoelastic properties regulate 2D and 3D migration remains complex, some general themes are emerging. In 2D, an important similarity between the materials (alginate, HA-collagen IPNs, and HA dynamic networks) is that cell spreading is regulated by adhesivity, stiffness, and rate of stress relaxation. For example, in the setting of sufficiently high adhesive density, cells are able to spread more on stiff, fast-relaxing surfaces than on their elastic counterparts. These regulatory relationships are even more poorly understood in 3D, where much energy has focused on how incorporating viscoelasticity into matrices can help overcome confinement limitations. Specifically, plastic deformation is vital to open migratory paths. While plasticity is pertinent for 3D migration, its role in 2D migration is not fully understood. Of course, these themes are very broad, and our understanding of the underlying regulatory relationships is likely to change as new materials are characterized.

## Conclusion

While the field’s consideration of biomaterial mechanics has traditionally focused on elastic properties, the importance of viscous properties is becoming increasingly appreciated. In this review, we have discussed the origin and significance of viscous properties in native and engineered hydrogel materials. We have also discussed how viscous properties can influence cell migration in 2D and 3D, which has prompted fresh examination of relationships derived from purely elastic materials. An important future challenge will be to better integrate these lessons into engineered materials, especially in fibrous materials that have the capability of mimicking fibrous matrices found in tissue.

## Statement of Significance

Most tissues and many biomaterials exhibit some viscous character, a property that is increasingly understood to influence cell behavior in profound ways. This review discusses the origin and significance of viscoelastic properties of common biomaterials, as well as how these cues are processed by cells to influence migration. A deeper understanding of the mechanisms of viscoelastic behavior in biomaterials and how cells interpret these inputs should aid the design and selection of biomaterials for specific applications.
